# Retraction Note: Facile design of an ultra-thin broadband metamaterial absorber for C-band applications

**DOI:** 10.1038/s41598-021-81808-1

**Published:** 2021-01-19

**Authors:** Nguyen Thi Quynh Hoa, Tran Sy Tuan, Lam Trung Hieu, Bach Long Giang

**Affiliations:** 1 School of Engineering and Technology, Vinh University, 182 Le Duan, Vinh City, Vietnam; 2 NTT Hi-Tech Institute, Nguyen Tat Thanh University, Ho Chi Minh City, Vietnam

Retraction of: *Scientific Reports* 10.1038/s41598-018-36453-6, published online 24 January 2019

The authors are retracting this Article.

We were alerted to the possibility that our designed structure is a broadband cross-polarization metamaterial converter, rather than a broadband metamaterial absorber.

We have now performed additional simulations and calculations, confirming that we did not account for cross-polarized reflection in the absorption calculation.

As such, we are unable to support the conclusions regarding design of a broadband metamaterial absorber, because the designed structure exhibits broadband cross-polarization.

All authors agree to the retraction of the Article.

